# Cutaneous Leukocytoclastic Vasculitis Following Influenza Vaccination in Older Adults: Report of Bullous Purpura in an Octogenarian after Influenza Vaccine Administration

**DOI:** 10.7759/cureus.2323

**Published:** 2018-03-14

**Authors:** Stella X Chen, Philip R Cohen

**Affiliations:** 1 School of Medicine, University of California, San Diego; 2 Department of Dermatology, University of California, San Diego

**Keywords:** adults, bullous, cutaneous, influenza, leukocytoclastic, older, purpura, vaccination, vaccine, vasculitis

## Abstract

The influenza vaccination is recommended annually for protection against influenza infection. Adults over 65 years of age are especially vulnerable to complications from influenza infection; in addition, they constitute the largest group of influenza vaccination recipients each year. Cutaneous leukocytoclastic vasculitis involves inflammation of small vessel walls by neutrophils. An 88-year-old man with a history of idiopathic pulmonary fibrosis who developed bullous cutaneous leukocytoclastic vasculitis 14 days after receiving the influenza vaccine is described and the characteristics of influenza-associated cutaneous leukocytoclastic vasculitis in older individuals are reviewed.

## Introduction

The influenza vaccine is recommended worldwide for persons aged six months and older, with exigence emphasized for populations most vulnerable to influenza morbidity such as elderly individuals over 65 years of age [1]. Influenza vaccination is the most effective method for reducing transmission, acquisition, and complications of seasonal influenza; however, the adverse effects of influenza vaccination in the elderly population are not well-studied [1]. Cutaneous leukocytoclastic vasculitis, characterized by neutrophilic inflammation of the vessel walls in the skin, most commonly manifests as lower extremity palpable purpura [2]. An 88-year-old man who developed bullous cutaneous leukocytoclastic vasculitis of the upper and lower extremities following influenza vaccination is described and the characteristics of influenza vaccine-associated cutaneous leukocytoclastic vasculitis in older adults are reviewed.

## Case presentation

**Figure 1:**
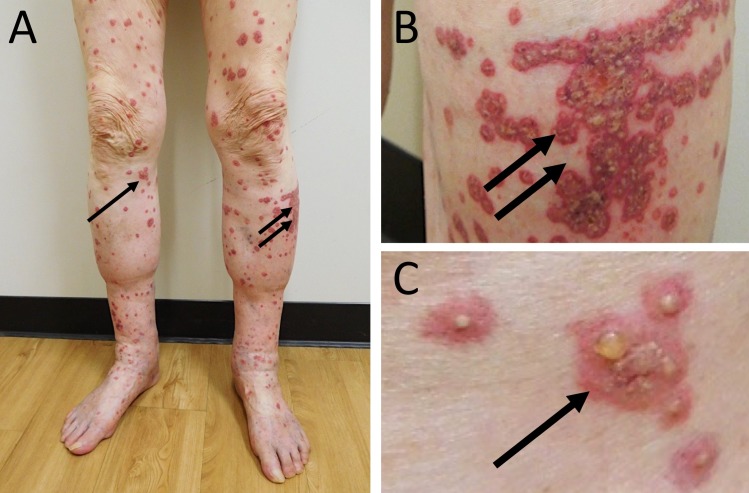
Bullous cutaneous leukocytoclastic vasculitis on the lower extremities. Distant (A) and closer (B and C) views of pruritic, erythematous-to-violaceous plaques with central vesicles (arrows) on the lower extremities of an 88-year-old Caucasian man who developed cutaneous leukocytoclastic vasculitis two weeks after receiving an influenza vaccination.

**Figure 2:**
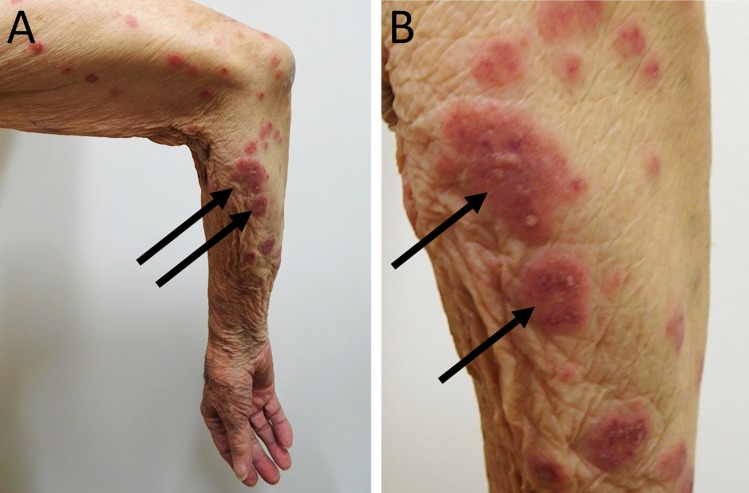
Bullous cutaneous leukocytoclastic vasculitis on the upper extremities. Distant (A) and closer (B and C) views of pruritic, erythematous-to-violaceous plaques with central vesicles (arrows) that appeared on the right upper extremity of an 88-year-old Caucasian man 14 days after receiving an intradermal influenza vaccination.

An 88-year-old Caucasian man with a history of gastroesophageal reflux disease, heart failure, hypertension, and idiopathic pulmonary fibrosis presented with a sudden eruption of pruritic, erythematous-to-violaceous purpura and plaques with central pustules distributed along the lower and upper extremities (Figures 1, 2). The purpuric bullous lesions ranged from 5 millimeters to several centimeters and had not been present at a clinic visit three weeks prior. He denied fever, arthralgia, difficulty breathing, swelling, anuria, or other systemic symptoms. He had no prior history of allergy or purpuric skin eruption and had not recently started any new medication. The man revealed that two weeks prior he had received his annual intradermal influenza vaccination. He denied any complications with previous influenza vaccines.

Biopsies obtained from the left thigh and left arm both showed similar findings: neutrophils and fragmented neutrophil nuclei within and surrounding the walls of the superficial vessels with fibrin deposition within the vessel walls. There was pronounced edema in the papillary dermis and a collection of neutrophils in the overlying epidermis. Correlation of the patient’s clinical presentation, pathology findings, and recent influenza vaccination established a diagnosis of bullous cutaneous leukocytoclastic vasculitis triggered by influenza vaccination.

**Figure 3:**
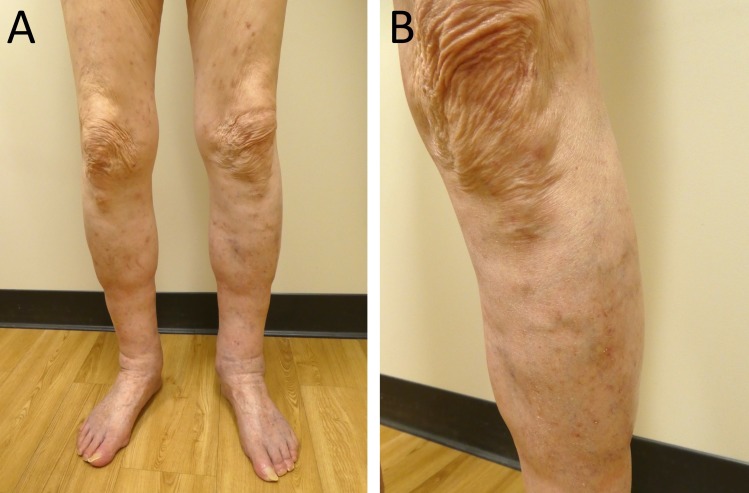
Resolution of bullous cutaneous leukocytoclastic vasculitis on the lower extremities. Distant (A) and closer (B) views of resolved influenza vaccine-associated bullous cutaneous leukocytoclastic vasculitis following 12 days of oral prednisone and topical triamcinolone cream on the lower extremities of an 88-year-old Caucasian man.

Other organs, such as the kidneys, may be associated with vasculitis; however, our patient refused additional laboratory testing. He was started on 60 milligrams of oral prednisone (which was tapered over the next 11 days and stopped) and topical triamcinolone 0.1 percent cream twice daily. At a follow-up visit 12 days later, examination showed that all of his skin lesions had resolved (Figure 3).

## Discussion

The influenza vaccine is recommended annually to individuals six months and older to prevent development, transmission, and complications of seasonal influenza. Increased efforts are focused on vaccinating populations at higher risk of influenza morbidity including pregnant women, children under five years of age, adults 65 years and older, and individuals who are immunosuppressed or have chronic medical conditions [1].

Elderly individuals over 65 years of age continue to be the largest age group to receive influenza vaccination. In addition, they are the group of patients with the greatest morbidity associated with influenza. Therefore, it is important to define, as best as possible, the potential complications arising from influenza vaccination in this population. However, more studies are needed to better characterize the adverse effects of influenza vaccines in elderly individuals [1].

The most common adverse effects associated with influenza vaccination are injection site reactions (such as local erythema and soreness), headache, fever, nausea, and myalgias. Very rarely, cutaneous leukocytoclastic vasculitis may occur [1].

Cutaneous leukocytoclastic vasculitis—also referred to as cutaneous vasculitis, hypersensitivity vasculitis, and cutaneous small-vessel vasculitis—is pathologically characterized by inflammation of the small-vessel walls by infiltrating neutrophils. It affects 30 million individuals per year with equal distribution between men and women. It most commonly occurs in superficial post-capillary venules, but may also involve larger and deeper vessels [2].

The typical presentation of cutaneous leukocytoclastic vasculitis is palpable purpura on the lower extremities that may be pruritic, painful, or burning. Other morphological presentations include ulcers, pustules, or vesicles. Nodules or livedo reticularis can occasionally be seen when medium-sized or deeper vessels are affected. Systemic symptoms—including fever, chills, arthralgias, and malaise—may also occur [2].

The diagnosis of cutaneous leukocytoclastic vasculitis is typically confirmed with a skin biopsy. Pathognomonic features, observed on microscopic examination, include fibrin deposition, fibrinoid necrosis with neutrophil infiltration of the vessel walls, fragmented neutrophil nuclei, and extravasated erythrocytes. The specific mechanism of pathogenesis with regards to influenza vaccine-associated leukocytoclastic vasculitis remains to be determined. However, the etiology of cutaneous leukocytoclastic vasculitis is thought to involve circulating immune complexes [2]. Hence, influenza vaccine-related antigens may promote the development of antibodies to which they subsequently bind; thereafter, the antigen-antibody complexes target affected organs such as the vascular structures of the skin.

Cutaneous leukocytoclastic vasculitis is most commonly idiopathic; however, it may be associated with infection (15 to 20 percent), autoimmune and connective tissue disorders (15 to 20 percent), new medications (10 to 15 percent), and malignancy (five percent). Rarely, vaccinations—including the influenza vaccine—can trigger cutaneous leukocytoclastic vasculitis [2,3].

Including this report, 11 cases of cutaneous leukocytoclastic vasculitis following influenza vaccination have been documented in elderly individuals 60 years and older (Table 1) [4-10]. These include six women and five men between the ages of 60 and 89 years (median age of 71 years). The women ranged in age from 60 to 89 years (median age of 75 years) and the men ranged in age from 60 to 88 years (median age of 70 years).

**Table 1:** Clinical characteristics of 11 individuals with cutaneous leukocytoclastic vasculitis following influenza vaccination. (A) A, age (in years); C, case; Clob, clobestasol proprionate ointment; CR, current report; CS, corticosteroids; DM2, type 2 diabetes; F, female; IPF, idiopathic pulmonary fibrosis; Kid, kidneys; HD, hemodialysis; HTN, hypertension; M, male; MDS, myelodysplastic syndrome; ND, not described; O, onset after vaccination (in days); OOI, other organs involved; Ova Ca, ovarian carcinoma; PMH, past medical history; PR, prior reaction after influenza vaccination; Pred, prednisone; Predl, prednisolone; Ref, reference; RT, recovery time (in weeks); S, sex; TAC, triamacinolone cream; Tx, treatment. (B) An 89-year-old woman developed palpable purpura and erythematous-to-violaceous patches of the lower extremities twice, both times following administration of an influenza vaccination. In both instances, her rash self-resolved. (C) An 88-year-old man developed pruritic erythematous-to-violaceous plaques with central pustules involving the upper and lower extremities. No additional testing was done to study other organ involvement as the patient refused further testing.

C	A S	PMH	Presentation	OOI	PR	O	Tx	RT	Ref.
1	60 F	ND	Fulminant bullous necrotizing vasculitis involving lower extremities	None	None	10	Predl	12	[4]
2	60 M	IPF	Arthralgias, chills, fever; erythematous plaques and petechiae involving face, upper and lower extremities, and trunk	None	None	5	Pred	0.6	[5]
3	63 M	ND	Arthralgia, myalgias, and purpura involving lower extremities	Kid	ND	ND	Pred HD	6	[6]
4	70 M	MDS	Purpuric dermatitis involving trunk, upper and lower extremities, and back; nausea, diarrhea, anuria	Kid	ND	2	CS	2	[7]
5	71 F	ND	Painful purpura involving lower extremities	Kid	None	14	Predl	10	[8] C2
6	71 M	None	Arthralgia, fever, malaise, myalgia; pruritic, palpable purpura and pitting edema in buttocks and lower extremities	Kid	None	10	Pred	2	[9]
7	74 F	ND	Painful, bullous, and necrotic purpura involving lower extremities	Kid	None	7	Predl	40	[8] C1
8	76 F	ND	Purpura involving lower extremities	Kid	None	14	Clob	2	[8] C3
9	79 F	Ova Ca	Purpura involving lower extremities	Kid	None	14	Clob	12	[8] C4
10	88 M	IPF	Pruritic erythematous-to-violaceous purpuric plaques with central pustules involving upper and lower extremities	None^C^	None	14	Pred TAC	1.7	CR
11	89 F	DM2 HTN	Palpable purpura and nonblanching erythematous-to-violaceous patches involving lower extremities; pancytopenia	None	Yes^B^	11	None	3	[10]

One man was in the process of receiving immunosuppressive treatment for myelodysplastic syndrome [7], one woman was coincidentally diagnosed with ovarian carcinoma at the time of cutaneous leukocytoclastic vasculitis presentation [8], and two men (including our case) had histories of chronic idiopathic pulmonary fibrosis [5]. The cutaneous eruptions developed between two and 14 days after influenza vaccination, with a median latency period of 10 days. All individuals presented with purpura involving the lower extremities [4-10]; in addition, seven developed renal involvement [6-9], three reported arthralgia [5,6,9], and one had pancytopenia [10].

An 89-year-old woman had presented the preceding year with an episode of cutaneous leukocytoclastic vasculitis with no attributable cause at the time [10]. One year later, when she developed a similar eruption, it was then determined that both manifestations occurred 11 days after influenza vaccination. In all other cases, the individuals had received prior influenza immunizations without complications.

In general, most cases of cutaneous leukocytoclastic vasculitis are self-limited. Patients with limited skin involvement and no other symptoms may be treated with avoidance or removal of the inciting cause in addition to rest, leg elevation, and ice packs to affected regions. Patients who also develop arthralgia or widespread skin involvement often respond well to a short course of systemic corticosteroids [2]. In the case of our patient, he was recommended to avoid influenza vaccinations in the future to avoid the risk of repeated reaction.

Systemic corticosteroids were provided to eight patients with influenza-associated cutaneous leukocytoclastic vasculitis. Two patients were only treated with a high-potency topical corticosteroid: clobetasol proprionate 0.05 percent ointment [8]. Our patient received both systemic and topically administered corticosteroids.

One patient’s lesions resolved in three weeks without treatment [10]. Another patient developed acute renal failure requiring nine days of hemodialysis [6]. All patients experienced resolution of skin lesions and other organ involvement. The median recovery time was three weeks.

In some instances, recovery time was rapid despite dramatic skin involvement. A 60-year-old man with widespread petechiae and erythematous plaques involving the face, trunk, and extremities experienced resolution of his skin lesions within four days after initiating prednisone treatment. In contrast, two women with tender, necrotizing, and bullous purpura did not experience resolution of their symptoms until three and ten months later [4,8].

The prognosis of influenza vaccine-associated cutaneous leukocytoclastic vasculitis is favorable; however, evaluation for concurrent involvement of internal organs or associated systemic disorders should be considered. Indeed, although one of the patients with cutaneous leukocytoclastic vasculitis following influenza vaccination—a 63-year-old man—ultimately recovered without lasting sequelae, he required nine days of hemodialysis due to acute renal failure. Clinicians should consider additional laboratory testing including complete blood cell counts with differential, creatinine level, liver function tests, erythrocyte sedimentation rate, and urinalysis [2]. In our patient, further evaluation was not performed since he refused additional testing.

The influenza vaccination is the most effective method for preventing the acquisition and transmission of the influenza virus. As it continues to be increasingly used, particularly in older individuals, clinicians should be aware of cutaneous leukocytoclastic vasculitis as a potential adverse effect of influenza vaccination. In instances of cutaneous leukocytoclastic vasculitis without ostensible attributable causes, such as in our patient, the affected individual should be asked about recent vaccination history.

The possibility of influenza-related cutaneous leukocytoclastic vasculitis should be entertained in patients with chronic medical conditions such as idiopathic pulmonary fibrosis which was observed in two of 11 individuals reported (Table 1). Similarly, patients who are immunosuppressed, such as the 70-year-old man receiving treatment for myelodysplastic syndrome [7] and the 89-year-old man who was concurrently found to have pancytopenia [10], may be at higher risk of developing leukocytoclastic cutaneous vasculitis. Finally, malignancy may also increase susceptibility to cutaneous leukocytoclastic vasculitis following vaccination [7,8]. While it is possible that the development of cutaneous leukocytoclastic vasculitis in these patients may be coincidental, it will be important to further characterize the incidence and types of complications associated with influenza vaccination in the older population.

## Conclusions

Older individuals—particularly those who are at least 65 years of age—are especially vulnerable to influenza infection complications and should receive an annual influenza vaccination. To date, there have been 11 cases of older individuals, including an 88-year-old man with idiopathic pulmonary fibrosis presented here, who have developed cutaneous leukocytoclastic vasculitis following influenza vaccination. Comorbidities which may have contributed to cutaneous leukocytoclastic vasculitis susceptibility in older individuals who received influenza vaccination include malignancy, immunosuppression, and chronic medical conditions such as pulmonary fibrosis. Clinicians should consider cutaneous leukocytoclastic vasculitis as a possible complication in older individuals who receive the influenza vaccination.
